# Far from Home: Managing Incidental Findings in Field Research with Portable MRI

**DOI:** 10.1017/jme.2024.169

**Published:** 2024

**Authors:** Susan M. Wolf, Judy Illes

**Affiliations:** 1: UNIVERSITY OF MINNESOTA, MINNEAPOLIS, MN, USA; 2: UNIVERSITY OF BRITISH COLUMBIA, VANCOUVER, BC, CANADA

**Keywords:** Incidental Findings, Portable MRI, Field Research, Underserved Populations, Neuroimaging, Research Ethics

## Abstract

Portable MRI for neuroimaging research in remote field settings can reach populations previously excluded from research, including communities underrepresented in current brain neuroscience databases and marginalized in health care. However, research conducted far from a medical institution and potentially in populations facing barriers to health care access raises the question of how to manage incidental findings (IFs) that may warrant clinical workup. Researchers should not withhold information about IFs from historically excluded and underserved population when members consent to receive it, and instead should facilitate access to information and a pathway to clinical care.

## Introduction

Portable Magnetic Resonance Imaging (pMRI) for neuroimaging research in remote field settings holds great potential to reach populations previously excluded from research, including minoritized and underserved communities underrepresented in current neuroscience databases.^1^ However, research conducted far from a medical institution and in populations that may not have adequate health coverage or established primary care relationships raises the question of how to manage incidental findings (IFs). These findings may warrant clinical work-up, sometimes urgently. Many prior guidelines on return of IFs to research participants condition return of results (RoR) and IFs on clinical actionability.^2^ However, in a population with poor access to clinical care, some commentators have questioned whether IFs are actionable as a practical matter and have suggested reduced or no return of IFs to research participants.^3^ Other commentators have argued that historically disadvantaged participant communities have an equal or greater entitlement to their results and IFs.^4^ With the emergence of pMRI facilitating neuroscience research in such communities, this debate cannot be avoided.

Prior ethical guidance has addressed return of results and IFs (or secondary findings, which we include under IFs in this discussion of neuroimaging research^5^) when a subset of participants may lack health care coverage and established care relationships; the dominant recommendation is to anticipate this problem and establish a pathway to referral and clinical workup.^6^ Some studies may pay for that initial clinical workup as part of the research budget, though they will typically not pay for subsequent care.^7^ However, moving the locus of neuroimaging research outside the hospital setting to field locations that may be hundreds of miles from a medical home raises the possibility that most or all of the research participants have been underserved and may lack health care coverage and a medical home. Some pMRI research will predictably raise this issue.^8^ Indeed, pMRI research in remote settings may involve data transfer to a cloud platform, identification of IFs enabled by artificial intelligence (AI), and interpretation by researchers and clinicians who are far from the location where scanning was conducted. Dealing with these issues in pMRI research in remote and underserved field settings thus requires ethical analysis and a plan for addressing these problems in partnership with the participant community. Researchers will need to articulate the agreed plan in their research proposal and protocol, and in seeking an adequate research budget.

The goal of this paper is to analyze the question of how to approach IFs in pMRI research in remote field settings and generate initial recommendations. Our focus here is on IFs identified by structural MRI, as there is currently less consensus on what would constitute an IF in functional imaging with MRI.

The goal of this paper is to analyze the question of how to approach IFs in pMRI research in remote field settings and generate initial recommendations. Our focus here is on IFs identified by structural MRI, as there is currently less consensus on what would constitute an IF in functional imaging with MRI.^9^ Decades of work on managing IFs emphasize the inevitability of IFs in neuroimaging — with varying frequency and significance, depending on the age of the population and recruitment criteria — and the necessity of advance planning and transparency. We argue here that historically underserved populations that may be reached by pMRI research deserve no less: research in these populations warrants careful community consultation to devise an acceptable plan, consideration of the potential impact of an incidental finding in the context of inadequate health care coverage, and a focus on research that avoids exploitation and instead confers local value including by offering information of potential health importance.^10^

## I. Past Work on Incidental Findings

The earliest writings on IFs from imaging the brain focused on disclosure based on urgency, with clinical actionability the dominant criterion used to determine whether to return an unexpected finding to a research participant.^11^ Subsequent work took a more holistic perspective, considering data on IF incidence^12^ and the perspectives of potential recipients,^13^ as well as age and cultural factors,^14^ in an effort to develop recommendations and protocols. As consideration of offering IFs as well as research results to participants has unfolded over more than 25 years of work, criteria discussed to determine what findings to offer research participants have expanded to consider findings of potential personal importance (whether or not the findings are clinically actionable in the sense that they could be used by clinicians to alter diagnosis or care), and to contemplate offering all findings in some studies. Examples of potential personal importance can include reducing anxiety, enabling participants to communicate with family members, and informing decisions about health and life insurance.^15^

The literature on return of results and IFs is now extensive and ranges from normative analyses to empirical studies, policy reports, and case studies. In the context of neuroimaging research, that literature generally converges on three principles: (1) findings in structural brain imaging can indicate pathology and may require urgent clinical attention, (2) increased imaging resolution will yield increased numbers and types of IFs, and (3) it is essential to establish a pathway for managing IFs in the earliest phases of research design, with appropriate provision for informed consent, expert consultation to evaluate potential IFs, and effective referral to clinical care when clinical pursuit of the IF is warranted. The introduction of pMRI capabilities take the already complex discussion of how to manage IFs to a new level: field strengths vary for pMRI, the support of a well-resourced medical center may not be available when pMRI scanning is conducted in a rural and remote community, and preferences for management of IFs as well as cultural norms for control of data may vary by participant community.

Based on past empirical and theoretical research, procedural approaches to identification of IFs in research may take at least four forms: (1) no screening for IFs, and no offer of any findings; (2) no screening for IFs, but offering findings of potential importance that are accidentally discovered; (3) systematic review of those scans on which a suspected anomaly is spotted; or (4) routine review of all scans for findings of potential importance.^16^ Similarly, there is a range of substantive approaches to defining the set of IFs to be offered to research participants: (A) offering none; (B) offering only IFs suspected to be clinically actionable; (C) offering a broader set of IFs, including those of potential personal importance to research participants even if not clinically actionable; and (D) offering all findings. **Table 1** depicts likely combinations of screening strategies and scope decisions.^17^

**Table 1:**
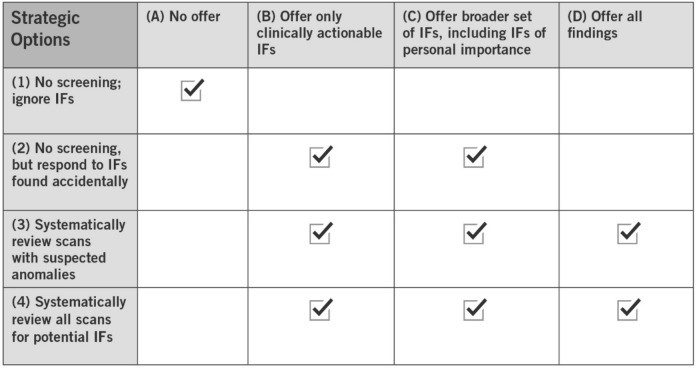
Combinations of procedural approaches to identifying IFs (vertical axis) and substantive approaches to defining the scope of IFs to offer (horizontal axis) are marked with a check.

The trend has been away from options (1) and (A) — ignoring IFs and offering none — as studies overwhelmingly show participant interest in receiving them and ethical duties including respect and reciprocity have been seen to weigh in favor of identifying and offering these findings to participants.^18^ For example, a study by Kirschen et al. demonstrated overwhelming receptivity to disclosure of unexpected neuroimaging results regardless of expected degree of clinical actionability.^19^ A study by Wilkins and colleagues has argued that the scope of findings offered to research participants should be based not on clinical actionability, but on what information the participants value, which may extend beyond IFs and return of results to additional insights generated by the study.^20^ Based on a national survey of a diverse U.S. sample to ascertain their views on return of research results, those authors “found that participants across all demographics highly valued receiving information from research studies and were more likely to trust researchers and to volunteer if research information were returned.”^21^

In deciding what approach to take, researchers, their institutions, and review boards should consider participants’ right to control information about themselves, researcher duties to respect participant autonomy, and researchers’ ancillary care obligations.^22^ Those undertaking and overseeing pMRI research should also prioritize the imperative to collaborate with the participant community in planning and executing research, including planning for the management of IFs.^23^ Numerous commentators have argued that there is a moral duty to offer information on IFs to participants who contribute their time and brain data,^24^ that is, to return information about an IF that is identified, accompanied by referral to resources for further information and clinical follow-up.

However, the specific challenge this paper addresses is return of IFs in remote and underserved populations. McMahon and colleagues, analyzing researcher attitudes toward RoR in under-resourced settings, have questioned, “how the process of return would actually unfold, especially in under-resourced, under-served, and under-represented communities.”^25^ They note that, “[p]ersistent disparities in access to health care have raised additional questions of whether such results are, or can be, equitably incorporated into clinical care….”^26^ Graham and coauthors have expressed concern over return of IFs in health care systems that are publicly funded, where pursuing ambiguous IFs may consume scarce health care resources.^27^

## II. Ethical Duties in Underserved Participant Communities

Arguments made by other commentators that researchers owe less of a duty to ascertain and offer return of IFs in underserved and under-resourced participant communities turn on several claims: (1) that participants lack the resources to pursue clinical follow-up that would yield benefit, (2) that participants would thus experience more burden than benefit from return of IFs, and (3) that efforts to ascertain, return, and offer initial clinical assessment of whether further clinical care is needed would unreasonably deplete research resources, thereby disadvantaging those who stand to benefit from the research knowledge sought by the study. We consider each of these claims in turn.

The argument that individuals should not even be offered information of potential clinical importance if researchers have already concluded these individuals lack to resources to fund clinical follow-up that may yield benefit (the first claim) makes several questionable assumptions. It assumes that investigators looking at a research IF that has not yet undergone clinical evaluation already know the nature of the finding, what surveillance or interventions (if any) will be recommended, what course the patient will then choose, what costs are associated with that chosen course, and what funding options the patient will have (or be offered) to pursue that course. Of course, when an IF is initially identified in research, the investigator has little basis on which to make these assumptions. Nor will investigators generally have a sound basis for the second claim, that the participant will experience more burden than benefit. Individuals vary in what they consider a benefit and a burden. Moreover, the literature on return of research results and IFs recognizes a wide range of potential benefits, both clinical and non-clinical.^28^ These include knowledge about one’s risks and health, information that might motivate the individual to seek additional insight about the finding or to volunteer for a relevant clinical trial, and the opportunity to share the finding with family members or other loved ones. On the burden side, overwhelmingly studies on return of results and IFs have not found significant and lasting psychological harms, even when the finding presented to the participant indicates a serious and untreatable condition.^29^

When investigators fulfill their obligation to consult the participant community in co-designing the study^30^ — including co-designing the management of IFs — investigators have the opportunity to elicit community perspectives on what IFs should be offered, how initial clinical evaluation should proceed, and then how to offer a pathway to timely clinical care for those participants who need and choose it. The community can also work with the investigators to anticipate the types of IFs likely to arise in the planned study, in order to consider what pathways to care are needed and how they can be funded. Researchers should not deprive communities of the opportunity to collaborate on ensuring access to care for participants with IFs. On the contrary, investigators should partner with participant communities to assess what pathways will likely be needed and whether the research funder or others can help provide access. These investigator duties flow from obligations to avoid treating research participants as mere means, to avoid research that extracts knowledge from a community with no return of local benefit, and to respect the needs and preferences of participant communities, especially those communities that have historically been exploited or neglected in research.^31^

The third claim, that managing and returning IFs will deplete the research budget and reduce the ability to provide future benefit from research findings, is also problematic. A large literature has used a range of ethical arguments — including ancillary care, duty to warn, and reciprocity to research participants — to support researcher duties to manage IFs. These duties, like virtually all ethical duties that researchers shoulder, require resources and personnel effort. The fact that conducting research ethically takes time, effort, and resources is a truism, rather than an argument against those ethical responsibilities.

Some commentators have nonetheless questioned whether there is a limit to what proportion of the research budget should go to managing IFs, though there is no agreed formula or percentage limit.^32^ The problem with the question as often framed is its assumption that any resources spent managing IFs necessarily take away from resources to create new research knowledge. Duties to manage IFs responsibly devolve on both researchers and funders; research funders should consider enlarging the budget, if necessary to conduct the research ethically and in a way acceptable to the participant community (including with respect to managing IFs).^33^ A number of studies have included in their research an investigation of how best to manage return of results and IFs,^34^ so that resources devoted to managing IFs contribute to the knowledge gained. That knowledge may be particularly lacking and needed in historically underserved participant communities.^35^

Ultimately, participant communities that have been underserved and neglected in neuroscience research have at least as strong a claim to the return of IFs as other more resourced communities that have not been excluded. We do not adjudicate among the differing ethical theories and arguments supporting return of IFs; together they offer a robust ethical architecture that has supported a sea change toward a new normal of return of results and IFs.^36^ Against that background, we argue that if those participant populations that have more resources are offered IFs, then underserved and under-resourced populations should not be deprived of this information because of their historical and comparative disadvantage.^37^ Indeed, considerations of justice, equity, and respect give the claims of such populations added weight. Moreover, the imperative to include previously neglected populations in neuroscience research to diversify neuroscience data sets and ensure that increasing knowledge of the brain includes all populations^38^ requires developing approaches to managing IFs that address barriers to care in underserved communities.

This means that community consultation is key in resolving how to handle IFs in remote and under-resourced populations. Respect for participant and community preferences and careful attention to how they see the benefits and risks of different IF strategies are essential. This is especially the case for research involving participants who may not have a medical home, who have historically had poor access to medical care, and who may hold community-based and culturally specific views about wellness that differ from narrowly biomedical explanations, or who may have experienced past adverse relationships in prior research. Moreover, transparency is vital for remote field research with pMRI as it may involve data transfer to a cloud platform, AI screening, and potentially major geographical, financial, structural, and cultural barriers to access follow-up clinical care. We consider these variables next.

## III. Challenges in Managing IFs in Remote pMRI Research

Portable MRI research data collected in a rural or remote region will not reside locally in most cases. Instead, the data will likely be transferred to a cloud platform and ultimately to a research institution that is far from the point of collection.^39^ This situation is not unlike that in biobanks and archived data, which have become crucial engines of genetic and genomic research.^40^ However, because identification of IFs requires access to and analysis of data, a research study’s approach to IFs must be embedded in a broader approach to consent, data management, and control. This too requires community consultation and the development of a data management plan that is acceptable to the participants and community. Some communities may require that they maintain control of data — for example, through data sovereignty and data ownership^41^ — while others may negotiate shared governance or protection for participant data access and control.^42^ The data framework developed for the study will affect the flow of data and feasibility of any IFs plan.

When identification or reidentification of an individual participant is possible and the community agrees to a research design involving an offer of IFs to participants, investigators should ensure that they can: (1) establish a process for timely review of brain scans, (2) specify the criteria for identifying an IF for potential return to the participant, (3) reliably reidentify the individual participant, and (4) implement a process acceptable to the community for recontacting the participant (see **Table 2**). This is the case whether scans are screened using AI methods, by humans with radiology expertise, or both. AI is proving to have highly accurate diagnostic capabilities for many, but not all communities and conditions.^43^ As AI improves and bias is reduced with more inclusive training sets, the benefits of better detection of clinical actionable findings may well accrue to research in rural and remote locations, even for scanners with field strength that is limited to 0.5T.

**Table 2:** Summary of key steps for researchers to take in planning the management of incidental findings (IFs) for pMRI research in remote field settings, so that the plan can be incorporated into the research protocol.

Key Steps	Specific Tasks
**Consulting with community** on IFs strategy	Consult with the community about offering information about individual-specific IFs to participants, and whether participants should be offered different options for return of IFs, including the possibility to opt-out.
Working with the community to determine **plan specifics**	Consult with the community on the plan for reviewing scans, the scope of review, what findings will be offered to participants (see Table 1), reidentification of participants as appropriate to enable return of IFs, and workflow with relevant personnel (e.g., radiologists or neuroradiologists) for reviewing and communicating IFs.
Ensuring that **funding** and **support resources** are commensurate with the strategy	Work with funders and others to ensure that the strategy and plan specifics can be supported by the research budget, health system, individual participant or community, or some combination.
Designing the **consent process**	Design the consent process to inform potential participants of the IFs strategy and elicit their consent. Consider whether individual participants should be offered different options for return of IFs (e.g., what subset to return).
Creating a plan for **review of scans**	Establish a process for timely review of scans for IFs. Ensure appropriate personnel (including radiology or neuroradiology expertise), workflow, and resources.
Establish process for **offering IFs**	Establish a process for timely reidentification of participants to be offered return of IFs, and a workflow with appropriate personnel for offering and communicating IFs.
Creating a path for **initial clinical evaluation of IFs of possible** clinical significance	Establish a process for initial clinical evaluation of IFs, including transportation to local or distant site offering the expertise and capability to perform a clinical evaluation to determine if the finding warrants a transition to follow-up clinical care.
Creating a **referral pathway** for follow-up clinical care	Determine how to refer participants with IFs for further clinical care beyond initial evaluation. Consider how to help participants to secure access to care for IFs, especially those who lack public coverage, insurance, or other resources.
Ensuring appropriate **oversight and approvals**	Ensure appropriate oversight and approvals of the IFs plan, including from IRB and (where relevant) from other institutional and regulatory authorities.*

* Return of genomic IFs and results, for example, has involved the U.S. Food and Drug Administration’s Investigational Device Exemption (IDE) process. See, e.g., E. Venner et al., “Whole-genome Sequencing as an Investigational Device for Return of Hereditary Disease Risk and Pharmacogenomic Results as Part of the *All of Us* Research Program,” *Genome Medicine* 14 (2022): 34, doi: 10.1186/s13073-022-01031-z. The use of investigational neuroimaging devices may also trigger regulatory requirements for management of IFs.

While the flexibility of pMRI has great potential to democratize brain imaging research and advance much-needed inclusion in neuroscience research, conducting research in remote and underserved communities may challenge three dimensions of the workflow for IFs: researchers’ ability to ensure (A) timely review of scans by expert radiologists or neuroradiologists, (B) a timely offer of information and counseling concerning IFs to the affected research participants, and (C) participant access to follow-on clinical evaluation and care. We consider each of these in turn.

Ensuring timely review of scans — whether the researchers and community have agreed to narrow review (e.g., only scans where a concern has been noted) or broader review (e.g., routine review of all scans) — requires that the research team has engaged radiologists or neuroradiologists.^44^ Providing them with rapid access to scans may require a workflow markedly different from the process in conventional MRI research within a major hospital. If scanning is done in a remote setting,^45^ data captured will likely be transferred to a cloud platform for interrogation and interpretation.^46^ Creating a workflow attentive to IFs will take planning before scanning begins and should be reviewed by an oversight committee such as an institutional review board (IRB) with independent authority and relevant expertise. Because a subset of IFs may require urgent clinical evaluation, the timeliness of expert review is crucial. In remote field settings, timely screening may also be needed to avoid losing the participant to follow-up.

Because some IFs will require rapid clinical work-up, participants who have agreed to receive information about their IFs should be offered that information in a timely way. In addition, participant mobility and the risk of losing participants to follow-up call for a timely offer of information. This too will require careful planning, appropriate personnel, and funding.

Perhaps the biggest challenge will be determining how to offer timely clinical evaluation and a pathway to follow-up care when a participant has elected receipt of an IF requiring clinical attention. Even providing indicated clinical care outside of a research protocol can be difficult in remote and under-resourced populations. For example, Harding et al. interviewed and surveyed neurosurgeons, neurologists, and other specialists across Canada to assess their perspectives on patient access to neuromodulation for drug-resistant pain, epilepsy, mental health, and movement disorders in remote areas of the country.^47^ The mixed-methods study found discordance between the perceived imperative of providing possibly lifesaving interventions for the four conditions, and the likelihood that these interventions would be available.

As pMRI moves into research use, a range of responses may emerge to discovery of IFs requiring clinical attention. One option is to offer the participant transport to a clinical center that can provide the evaluation needed. While this may be an appropriate option in some circumstances, burdens may include the disruption of asking a participant to leave their community for travel to a potentially distant and unfamiliar site. In addition, air transport for urgent clinical evaluation may be costly for the research budget and impossible for the participant to pay.^48^ Much work is needed to understand the types of IFs that would need to be addressed by travel to a medical center, who would pay for the response, and the views of participants and their communities about the risks and benefits of transporting a person far from home for this purpose.

However, transporting the research participant to a clinical center is only one option. As pMRI research becomes more common in remote field settings, creating outposts offering clinical evaluation may be worth considering, especially for multiple research projects operating in overlapping areas. Portable MRI scanners with the demonstrated and approved capacity to provide clinical diagnosis could be situated strategically and used as needed. Even if the researchers are using a low-field or ultra-low-field scanner, cooperatively positioning scanners with proven diagnostic capacity may be part of the answer to the IFs challenge.

## IV. Responsibilities in Conducting pMRI Research in Field Settings

With pMRI research emerging now, the time is ripe to address these issues as IFs are sure to arise in the field research that pMRI allows. As the systems begin to roll out and hands-on experience is gathered, this is the moment for researchers and professional organizations to attend to both the benefits and challenges. Partnering with the participant community and collaboratively devising solutions will be key. For example, in research involving First Nations, Native American, or Alaskan Native populations, using principles and approaches grounded in both Western bioethics and Indigenous methods^49^ will be important to address the IFs issue responsibly, with full understanding of IFs that could be identified; potential opportunity and burden for research participants, their family, and the community; and collaborative problem solving. Community leaders, Elders, and community members should not only be consulted, but should be full partners in addressing these issues.

Where research participants face significant barriers to accessing clinical evaluation and care — whether those barriers are a function of geography, economic hardship, reduced access to clinicians and care facilities, or other obstacles — addressing the reality that neuroimaging may uncover IFs can catalyze advocacy for access and innovative strategies. Clinicians at major medical centers, as well as providers at nursing stations and medical facilities in or near communities where research may take place, must be prepared to receive research participants who may become prospective patients through pursuit of IFs. This requires planning for and with a community well before the protocol starts, evidence-based analysis of the likelihood of IFs in the cohort studied, as well as plans for any broader return of results. The alternative of giving up from the start and denying participants the opportunity to receive information that wealthier and more resourced participants would be offered raises serious equity concerns. Such refusal to address the IFs challenge would only deepen health inequity. Under-resourced participants would be contributing to neuroimaging research and knowledge while being systematically deprived of information they would receive if they had more resources.

The argument that IFs should not be offered to less-resourced participants or communities because they cannot afford to take clinical action similarly entrenches inequity and fails to consider access options. As noted above, some studies provide clinical evaluation on the research budget.^50^ Moreover, major studies (or smaller studies working in concert) may have the leverage to create pathways to clinical evaluation and care. In addition, research participants may value being offered their results and IFs for a wide range of reasons — not just to access clinical interventions to alter diagnosis and treatment. When researchers offer a participant desired information about their findings, the researchers are demonstrating respect for the preferences and values of that participant. Indeed, communities may refuse to allow researchers access unless the researchers agree to share such findings.^51^ Moreover, some legal frameworks will require researchers to offer participants access to their individual specific findings^52^ and HIPAA-covered entities in the United States have duties to provide a participant with their research data at their request.^53^

In neuroimaging research, the incidence of IFs may make the problem more tractable than it first appears, though incidence varies by population and age group. IFs are generally found in less than 1% in people who are under 21 years (although these tend to be the most serious findings when detected), the incidence of clinically actionable neuroimaging IFs remains low in the adult population, and despite the high percentage in people older than 65 years (as much as 50%) these IFs are generally age-appropriate and require only routine follow-up.^54^ However, large-scale prospective studies on the incidence of IFs in research participants from underserved and under-resourced remote settings are needed to form a complete picture of the scope of the IFs challenge.

This calls for more research in remote field settings. Such research will require partnership with these communities, including on the return of results and IFs. Federal, state, provincial, and private funders should support this work, including responsible management of IFs. Failure to fund and plan for IFs risks deepening health inequities and perpetuating the exploitation of underserved populations by conducting neuroscience research without offering IFs that would be routinely offered in other studies. Portable MRI research has enormous potential to make progress in addressing inequities and historical exclusion from research. It also offers the potential to use pMRI to bring clinical evaluation and care closer to those in settings far from major medical centers.

## Conclusion

Both bioethics and neuroscience are increasingly confronting past exploitation of marginalized communities and demonstrating a commitment to equity, justice, and respect.^55^ Brain imaging research using pMRI has the potential to significantly broaden research participation and create more representative neuroscience databases yielding a fuller understanding of human diversity. The World Health Organization (WHO) advises that neurological disorders are the leading cause of disability worldwide and the second leading cause of death globally, accounting for approximately 9 million deaths per year.^56^ Ensuring attention to underserved populations and analysis of the disorders those populations face is essential to progress.

Breakthroughs in discovery research can directly impact the translational care pipeline. When studies are conducted in historically underserved communities and lead to therapeutic interventions, the likelihood that they will be applied in those communities is greater today than ever. Portable MRI will enable that. Health outcomes may not immediately improve in areas where medical care is lacking, but an increase in research on health that is inclusive and culturally competent in such areas will foster trust where it has not existed in the past or has been squandered. Making return of IFs work effectively in field research with historically neglected communities — with respect for local perspectives and priorities, creation of a feasible and supportive pathway to care, individual participant choice, and robust protections for participant confidentiality and data control — is a crucial next step.

## References

[1] The authors recognize their positionality as urban academic scholars who have participated in past research projects involving participants from traditionally underserved communities that are underrepresented in neuroscience databases, but who are not themselves members of those communities. Recommendations in this chapter are intended to offer initial guidance on managing the challenges that relate to IFs and the questions that any researcher-community team should jointly ask and resolve before embarking on a study in a rural or remote area.

[2] For discussion of the concept of actionability in return of results and IFs, see, e.g., S.M. Wolf and R.C. Green , “Return of Results in Genomic Research Using Large-Scale or Whole Genome Sequencing: Toward a New Normal,” Annual Review of Genomics and Human Genetics 24 (2023): 393–414; J. Illes et al., “Working Group on Incidental Findings in Brain Imaging Research. Ethics: Incidental Findings in Brain Imaging Research,” *Science* 311, no. 5762 (2006): 783–784; J. Illes et al., “Practical Approaches to Incidental Findings in Brain Imaging Research,” *Neurology* 70, no. 5 (2008): 384–390. Not all guidelines limit return of IFs to those deemed clinically actionable. See Wolf and Green, *supra*.

[3] See A. Ortiz-Osorno , L.A. Ehler , and J. Brooks , “Considering Actionability at the Participant’s Research Setting Level for Anticipatable Incidental Findings from Clinical Research,” Journal of Law, Medicine & Ethics 43, no. 3 (2015): 619–632; C.E. McMahon et al., “Interrogating the Value of Return of Results for Diverse Populations: Perspectives from Precision Medicine Researchers,” *AJOB Empirical Bioethics* 15, no. 2 (2024): 108–119, at 117. The authors of the latter article report empirical analysis of researcher perspectives, but their discussion section then concludes, “Failing to account for the structural inequities in diverse populations’ ability to access, apprehend, and act on [return of results] raises fundamental concerns about the approach to, and depth of, PMR’s commitment to diversity and inclusion. Moreover, such over-promise may further erode trust….” They urge looking for alternatives to return of results. 10.1111/jlme.12304 26479571

[4] See, e.g., H.K. Sullivan and B.E. Berkman , “Incidental Findings in Low-Resource Settings,” Hastings Center Report 48, no. 3 (2018): 20–28; C.A. Stewart et al., “Pragmatic Clinical Trial-Collateral Findings: Recognizing the Needs of Low-Resource Research Participants,” *American Journal of Bioethics* 20, no. 1 (2020): 19–21; M.B. Raymond et al., “Practices and Attitudes Toward Returning Genomic Research Results to Low-Resource Research Participants,” *Public Health Genomics* 24, no. 5–6 (2021): 241–252. A substantial literature discusses duties of ancillary care and researcher responsibilities to return results and IFs in resource-challenged international settings. See, e.g., H.S. Richardson and L. Belsky, “The Ancillary-Care Responsibilities of Medical Researchers: An Ethical Framework for Thinking about the Clinical Care that Researchers Owe Their Subjects,” *Hastings Center Report* 34, no. 1 (2004): 25–33; D. Ralefala et al., “Do Solidarity and Reciprocity Obligations Compel African Researchers to Feedback Individual Genetic Results in Genomics Research?” *BMC Medical Ethics* 21, no. 1 (2020): 1–11. 33148222 10.1186/s12910-020-00549-4 PMC7640670

[5] The distinction between secondary findings and incidental findings has been much debated in the context of genomic research, where the usual contrast drawn is between a predetermined list of genes and variants to be ascertained *versus* unexpected findings. See Wolf and Green, *supra* note 2. In large-scale genomic sequencing studies, a secondary findings list can circumscribe what can otherwise be a wide field of potential IFs. However, this distinction between secondary findings and IFs has garnered less attention in imaging research, where the scan itself limits the field of findings to be interpreted and radiologist conventions for reporting observed pathology are well established.

[6] See, e.g., S.M. Wolf et al., “Managing Incidental Findings and Research Results in Genomic Research Involving Biobanks and Archived Dataset,” Genetics in Medicine 14, no. 4 (2012): 361–384. But see McMahon et al., *supra* note 3, at 109 (“Yet this raises further considerations around how the process of return would actually unfold, especially in under-resourced, under-served, and under-represented communities…. Persistent disparities in access to health care have raised additional questions of whether such results are, or can be, equitably incorporated into clinical care. Questions of health justice and responsibility continue to be raised where under-served populations face barriers to translating ROR into care.”) (citations omitted); C.P. Neuhaus and J.T. Crane, “Experiences at a Federally Qualified Health Center Support Expanded Conception of the Gifts of Precision Medicine,” *American Journal of Bioethics* 21, no. 4 (2021): 70–72, at 71 (“precision medicine research must confront the challenges of returning genetic results to patients who are un/underinsured…. Little consideration has been given to precision medicine research participants (or their families) who will struggle to receive care for potentially serious conditions they learn about through research participation.”). 22436882 10.1038/gim.2012.23 PMC3597341

[7] See S.M. Wolf et al., “Navigating the Research-Clinical Interface in Genomic Medicine: Analysis from the CSER Consortium,” Genetics in Medicine 20, no. 5 (2017): 545–553. 28858330 10.1038/gim.2017.137 PMC5832495

[8] Portable MRI research may also be conducted in underserved and under-resourced urban populations. However, those participants are likely to be physically closer to a major medical center — whether for the conduct of the study or response to IFs — though they may face other barriers to access aside from geographical distance. This paper focuses on participants facing barriers to access that include distance. Portable MRI promises to make research possible in such settings, but raises the challenging issues this article addresses.

[9] Functional MRI research may involve both structural and functional scanning. When structural scanning (whether alone or in an fMRI study) reveals an IF, we consider it here a structural IF. Indeed, most discussion of IFs in fMRI focuses on IFs identified in performing structural scans (not functional ones) in the context of an fMRI study. See, e.g., A. Soumaré et al., “Prevalence, Severity, and Clinical Management of Brain Incidental Findings in Healthy Young Adults: MRi-Share Cross-Sectional Study,” Frontiers in Neurology 12 (2021): 675244, 10.3389/fneur.2021.675244. In the future, however, there may be greater consensus on what constitutes abnormal function, allowing identification of functional IFs. Those are beyond the scope of this paper. For discussion of the challenges surrounding IFs in fMRI research, see, e.g., J. Illes et al., “Ethical and Practical Considerations in Managing Incidental Findings in Functional Magnetic Resonance Imaging,” *Brain and Cognition* 50, no. 3 (2002): 358–365; N. Scott et al., “Incidental Findings in Neuroimaging Research: A Framework for Anticipating the Next Frontier,” *Journal of Empirical Research on Human Research Ethics* 7, no. 1 (2012): 53–57. 22378134 PMC10460148

[10] See F.X. Shen et al., “Ethical, Legal, and Policy Challenges in Field-Based Neuroimaging Research Using Emerging Portable MRI Technologies: Guidance for Investigators and for Oversight,” Journal of Law and the Biosciences 11, no. 1 (2024): lsae008, 10.1093/jlb/lsae008. 38855036 PMC11157461

[11] G.L. Katzman , A.P. Dagher , and N.J. Patronas , “Incidental Findings on Brain Magnetic Resonance Imaging from 1000 Asymptomatic Volunteers,” JAMA 282, no. 1 (1999): 36–39. 10404909 10.1001/jama.282.1.36

[12] See, e.g., B.S. Kim et al., “Incidental Findings on Pediatric MR Images of the Brain,” American Journal of Neuroradiology 23, no. 10 (2002): 1674–1677; Y. Li et al., “Rates of Incidental Findings in Brain Magnetic Resonance Imaging in Children,” *JAMA Neurology* 78, no. 5 (2021): 578–587; D.E. Sunny et al., “Prevalence of Incidental Intracranial Findings on Magnetic Resonance Imaging: A Systematic Review and Meta-Analysis,” *Acta Neurochirurgica* 164 (2022): 2751–2765.12427622

[13] See, e.g., M.P. Kirschen , A. Jaworska , and J. Illes , “Subjects’ Expectations in Neuroimaging Research,” Journal of Magnetic Resonance Imaging 23, no. 2 (2006): 205–209; A.K.J. Oerleman et al., “Impact of Incidental Findings on Young Adult Participants in Brain Imaging Research: An Interview Study,” *European Radiology* 32, no. 6 (2022): 3839–3845. There is also an extensive literature on participant preferences in other domains of research, including genomics. See, e.g., D.F. Vears et al., “Return of Individual Research Results from Genomic Research: A Systematic Review of Stakeholder Perspectives,” *PLoS ONE* 16, no. 11 (2021): e0258646, 10.1371/journal.pone.0258646. 16416438 PMC1560341

[14] See, e.g., K.B. Vander Wyst et al., “Communicating Incidental and Reportable Findings from Research MRIs: Considering Factors Beyond the Findings in an Underrepresented Pediatric Population,” BMC Medical Research Methodology 21 (2021): 275, 10.1186/s12874-021-01459-8. Cf. E. Brief, J. Mackie, and J. Illes, “Incidental Findings in Genetic Research: A Vexing Challenge for Community Consent,” *Minnesota Journal of Law, Science & Technology* 13, no. 2 (2012): 541–558 (in genetic research). 34865631 PMC8647358

[15] P.L. Bacon et al., “The Development of a Preference-Setting Model for the Return of Individual Genomic Research Results,” Journal of Empirical Research on Human Research Ethics 10, no. 2 (2015): 107–120, at 107–08; Wolf and Green, *supra* note 2. 25742675 10.1177/1556264615572092

[16] Sometimes a fifth option is noted — perform a clinical scan on all research participants. However, this is uncommon. See M. Graham , N. Hallowell , and J. Savelscu , “A Just Standard: The Ethical Management of Incidental Findings in Brain Imaging Research,” Journal of Law, Medicine & Ethics 49, no. 2 (2021): 269–281, at 272 (noting the approach at the National Institutes of Health). 10.1017/jme.2021.38 PMC8242825 34924060

[17] Table 1 depicts key decisions, but not all decisions to be made in determining process and scope. For example, a report from the National Academies additionally focuses on the quality of the findings (for example, whether genomic findings were generated in a CLIA-compliant laboratory). See National Academies of Sciences, Engineering, and Medicine (NASEM), *Returning Individual Research Results to Participants: Guidance for a New Research Paradigm* (Washington, DC: National Academies Press, 2018). But see S.M. Wolf and B.J. Evans, “Return of Results and Data to Study Participants,” Science 362, no. 6411 (2018): 159–160; S.M. Wolf and B.J. Evans, “Defending the Return of Results and Data,” *Science* 362, no. 6420 (2019): 1255–1256; B.J. Evans and S.M. Wolf, “A Faustian Bargain that Undermines Research Participants’ Privacy Rights and Return of Results,” *Florida Law Review* 71, no. 5 (2019): 1281–1345. 34305361 PMC8302004

[18] See, e.g., NASEM, *supra* note 17; Wolf and Green, *supra* note 2.

[19] Kirschen et al., *supra* note 13.

[20] C.H. Wilkins et al., “Understanding What Information Is Valued by Research Participants and Why,” Health Affairs 38, no. 3 (2019): 399–407. 30830824 10.1377/hlthaff.2018.05046 PMC6706772

[21] *Id.*

[22] On ancillary care obligations, see, e.g., Richardson and Belsky, *supra* note 4; H.S. Richardson , “Incidental Findings and Ancillary-Care Obligations,” Journal of Law, Medicine & Ethics 36, no. 2 (2008): 256–270; H.S. Richardson, *Moral Entanglements: The Ancillary Care Obligations of Medical Researchers* (Oxford University Press, 2012); H.S. Richardson, “Can the Research Team-Participant Relationship Ground Ancillary-Care Obligations?,” *Ethics & Human Research* 45, no. 1 (2023): 2–14. 10.1002/eahr.500152 36691690

[23] See F.X. Shen et al., “Ethical Issues Posed by Field Research Using Highly Portable and Cloud-Enabled Neuroimaging,” Neuron 105, no. 5 (2020): 771–775; F.X. Shen et al., “Emerging Ethical Issues Raised by Highly Portable MRI Research in Remote and Resource-Limited International Settings,” *NeuroImage* 238 (2021): 118210, 10.1016/j.neuroimage.2021.118210; Shen et al., *supra* note 10. 34062266 PMC8382487

[24] See M. Sadatsafavi et al., “An Ounce of Prevention Is Worth a Pound of Cure: A Cost-Effectiveness Analysis of Incidentally Detected Aneurysms in Functional MRI Research,” Value in Health 13, no. 6 (2010): 761–769; Wolf et al., *supra* note 6; Wolf and Green, *supra* note 2. 20561317 10.1111/j.1524-4733.2010.00755.x PMC10517630

[25] McMahon et al., *supra* note 3, at 2 (citations omitted).

[26] *Id*. (citations omitted).

[27] Graham et al., *supra* note 16. But see N. Murphy and C. Weijer , “Grey Matter – The Problems of Incidental Findings in Neuroimaging Research,” Journal of Law, Medicine & Ethics 49, no. 2 (2021): 282–284. 10.1017/jme.2021.39 34924037

[28] See Wolf and Evans (2018), *supra* note 17; Sullivan and Berkman, *supra* note 4, at 24–25.

[29] See Wolf and Green, *supra* note 2; Sullivan and Berkman, *supra* note 4, at 24 (“People tend to assume that receiving information about incidental findings or positive genetic test results will be extremely harmful, even though literature on affective forecasting and studies of people who have undergone genetic testing suggest that these harms are relatively minor and transient.”).

[30] See Shen et al., *supra* note 10.

[31] See *id.*; Ortiz-Osorno et al., *supra* note 3 (stressing the importance of community consultation).

[32] Compare H.S. Richardson , “Gradations of Researchers’ Obligation to Provide Ancillary Care for HIV/AIDS in Developing Countries,” Health Policy and Ethics 97, no. 11 (2007): 1956–1961, at 1959 (“Ethically, the important question about monetary cost is whether it is low, serious, or heavy in relation to the research budget. It is not the absolute monetary figure that matters.”). 10.2105/AJPH.2006.093658 PMC2040358 17901449

[33] See, e.g., Vander Wyst et al., *supra* note 14, at 8 (recommending that funders “[a]llocate fund use when an incidental finding is discovered to assist in provision of follow-up care, particularly among underserved populations that lack access to specialty care and/or insurance.”). On a range of approaches to returning research results to low-resource research participants, see, e.g., Raymond et al., *supra* note 4. See also Sullivan and Berkman, *supra* note 4, at 25.

[34] See, e.g., Vander Wyst et al., *supra* note 14; R.C. Green et al., “The Clinical Sequencing Exploratory Research Consortium: Accelerating the Evidence-Based Practice of Genomic Medicine,” American Journal of Clinical Genetics 98, no. 6 (2016): 1051–1066. 10.1016/j.ajhg.2016.04.011 PMC4908179 27181682

[35] See Raymond et al., *supra* note 4.

[36] See Wolf and Green, *supra* note 2.

[37] See NASEM, *supra* note 17, at 84–85: “[R]eturn of individual results should not contribute to health disparities and inequities in health care or health research. Research participants should have the same access to research results regardless of their socioeconomic status or their ability to access follow-up care when results indicate medical attention may be needed.” See also Sullivan and Berkman, *supra* note 4, at 22 (“treating groups differently seems unfair, particularly when it would disadvantage groups that are already worse off because of lack of access to health care resources”).

[38] See Shen et al., *supra* note 10.

[39] See Shen et al. (2020), *supra* note 23; Shen et al. (2021), *supra* note 23.

[40] Wolf et al., *supra* note 6.

[41] See, e.g., “The First Nations Principles of OCAP®,” First Nations Information Governance Centre, https://fnigc.ca/ocap-training/ (last visited Mar. 17, 2024).

[42] See, e.g., Shen et al., *supra* note 10.

[43] See, e.g., O. Oren , B.J. Gersh , and D.L. Bhatt , “Artificial Intelligence in Medical Imaging: Switching from Radiographic Pathological Data to Clinically Meaningful Endpoints,” Lancet Digital Health 2 (2020): e486–e488, 10.1016/S2589-7500(20)30160-6; B. Wildman-Tobriner et al., “Missed Incidental Pulmonary Embolism: Harnessing Artificial Intelligence to Assess Prevalence and Improve Quality Improvement Opportunities,” *Journal of the American College of Radiology* 18, no. 7 (2021): 992–999; A.W. Moawad et al., “Artificial Intelligence in Diagnostic Radiology: Where Do We Stand, Challenges, and Opportunities,” *Journal of Computer Assisted Tomography* 46, no. 1 (2022): 78–90. But see, e.g., S. Agarwal et al., “Systematic Review of Artificial Intelligence for Abnormality Detection in High-volume Neuroimaging and Subgroup Meta-analysis for Intracranial Hemorrhage Detection,” *Clinical Neuroradiology* 33, no. 4 (2023): 943–956. 33328116

[44] One interesting effort to enlist the help of an online consortium of radiology readers is the recently formed Collective Minds, “Collective Minds Radiology,” https://www.cmrad.com/ (last visited June 12, 2024). However, it is not clear whether this type of effort can ensure timely review.

[45] See, e.g., S.C.L. Deoni et al., “Development of a Mobile Low-Field MRI Scanner,” Scientific Reports 12, no. 1 (2022): 5690, 10.1038/s41598-022-09760-2. 35383255 PMC8982311

[46] See Shen et al., *supra* note 10.

[47] L. Harding et al., “Mapping the Landscape of Equitable Access to Advanced Neurotechnologies in Canada,” Canadian Journal of Neurological Sciences 50, no. s1 (2023): s17–s25, 10.1017/cjn.2023.18. See also C.M. Flood, B. Thomas, and E. McGibbon, “Canada’s Primary Care Crisis: Federal Government Response,” *Healthcare Management Forum* 36, no. 5 (2023): 327–332; H. Shahaed et al., “Primary Care for All: Lessons for Canada from Peer Countries with High Primary Care Attachment,” *Canadian Medical Association Journal* 195, no. 47 (2023): e1628–e1636, https://doi.org/10.1503/cmaj.221824. PMC10172973 37160675

[48] In Canada, public support may be available. See, e.g., T.K. Young et al., “Patient Transportation in Canada’s Northern Territories: Patterns, Costs and Providers’ Perspectives,” Rural and Remote Health 19 (2019): 5113, 10.22605/RRH5113. 31128577

[49] See, e.g., M. Kovach, *Indigenous Methodologies: Characteristics, Conversations, and Contexts* (University of Toronto Press, 2002); S.A. Stevenson, “Toward a Narrative Ethics: Indigenous Community-Based Research, the Ethics of Narrative, and the Limits of Conventional Bioethics,” Qualitative Inquiry 22, no. 5 (2016): 365–376; J. Kotalik and G. Martin, “Aboriginal Health Care and Bioethics: A Reflection on the Teaching of the Seven Grandfathers,” *American Journal of Bioethics* 16, no. 5 (2016): 38–43; J. Bardill and N.A. Garrison, “New Words and Old Stories: Indigenous Teachings in Health Care and Bioethics,” *American Journal of Bioethics* 16, no. 5 (2016): 50–52. 10.1080/15265161.2016.1159762 27111372

[50] On incorporating workup of IFs into research budgets, see Wolf et al., *supra* note 7.

[51] See, e.g., R. Morello-Frosch et al., “Communicating Results in Post-Belmont Era Biomonitoring Studies: Lessons from Genetics and Neuroimaging Research,” Environmental Research 136 (2015): 363–372; K.E. Boronow et al., “DERBI: A Digital Method to Help Researchers Offer ‘Right-to-Know’ Personal Exposure Results,” *Environmental Health Perspectives* 125, no. 2 (2017): A27–A33. 28145870 10.1289/EHP702 PMC5289917

[52] See, e.g., Boronow et al., *supra* note 51.

[53] See Evans and Wolf, *supra* note 17.

[54] See, e.g., G.L. Katzman , A.P. Dagher , and N.J. Patronas , “Incidental Findings on Brain Magnetic Resonance Imaging from 1000 Asymptomatic Volunteers,” JAMA 281, no. 1 (1999): 36–39; Kim et al., *supra* note 12; J. Illes et al., “Ethical Consideration of Incidental Findings on Adult Brain MRI in Research,” *Neurology* 62, no. 6 (2004): 888–890; M.W. Vernooij et al., “Incidental Findings on Brain MRI in the General Population,” *New England Journal of Medicine* 357, no. 18 (2007): 1821–1828; P. Wangaryattawanich et al., “Incidental Findings on Brain Magnetic Resonance Imaging (MRI) in Adults: A Review of Imaging Spectrum, Clinical Significance, and Management,” *British Journal of Radiology* 96, no. 1142 (2023): 20220108, 10.1259/bjr.20220108. PMC9975529 35522780

[55] For efforts to make progress in bioethics, see, e.g., R. Fabi and D.S. Goldberg , “Bioethics, (Funding) Priorities, and the Perpetuation of Injustice,” American Journal of Bioethics 22, no. 1 (2022): 6–13; F. Fletcher et al., “Bioethics Must Exemplify a Clear Path Toward Justice: A Call to Action,” *American Journal of Bioethics* 22, no. 1 (2022): 14–16; F.E. Fletcher et al., eds., “A Critical Moment in Bioethics: Reckoning with Anti-Black Racism through Intergenerational Dialogue,” *Hastings Center Report* 52, no. S1 (2022), https://onlinelibrary.wiley.com/toc/1552146x/2022/52/S1 (last visited Mar. 18, 2024). For efforts to make progress in neuroscience, see, e.g., V.M. Dotson and A. Duarte, “The Importance of Diversity in Cognitive Neuroscience,” *Annals of the New York Academy of Science* 1464, no. 1 (2019): 181–191; A. Ramamoorthy et al., “Racial/Ethnic Differences in Drug Disposition and Response: Review of Recently Approved Drugs,” *Clinical Pharmacology & Therapeutics* 97, no. 3 (2015): 263–273; J. Nketia et al., “Towards a More Inclusive and Equitable Developmental Cognitive Neuroscience,” *Developmental & Cognitive Neuroscience* 52 (2021): 101014, https://doi.org/10.1016/j.dcn.2021.101014; A. Gilmore-Bykovskyi et al., “Traversing the Aging Research and Health Equity Divide: Toward Intersectional Frameworks of Research Justice and Participation,” *Gerontologist* 62, no. 5 (2022): 711–720. 10.1093/geront/gnab107 PMC9154232 34324633

[56] “Brain Health,” World Health Organization, https://www.who.int/health-topics/brain-health#tab=tab_2 (last visited Nov. 30, 2023).

